# Mahi‐mahi (Coryphaena hippurus) life development: morphological, physiological, behavioral and molecular phenotypes

**DOI:** 10.1002/dvdy.27

**Published:** 2019-04-05

**Authors:** Prescilla Perrichon, John D. Stieglitz, Elvis Genbo Xu, Jason T. Magnuson, Christina Pasparakis, Edward M. Mager, Yadong Wang, Daniel Schlenk, Daniel D. Benetti, Aaron P. Roberts, Martin Grosell, Warren W. Burggren

**Affiliations:** ^1^ Department of Biological Sciences University of North Texas Denton Texas; ^2^ Department of Marine Ecosystems and Society University of Miami, Rosenstiel School of Marine and Atmospheric Science Miami Florida; ^3^ Department of Environmental Sciences University of California Riverside California; ^4^ Department of Marine Biology and Ecology University of Miami, Rosenstiel School of Marine and Atmospheric Science Miami Florida

**Keywords:** behavior, development, life span, mahi‐mahi, molecular biology, physiology

## Abstract

**Background:**

Mahi‐mahi (Coryphaena hippurus) is a commercially and ecologically important fish species that is widely distributed in tropical and subtropical waters. Biological attributes and reproductive capacities of mahi‐mahi make it a tractable model for experimental studies. In this study, life development of cultured mahi‐mahi from the zygote stage to adult has been described.

**Results:**

A comprehensive developmental table has been created reporting development as primarily detailed observations of morphology. Additionally, physiological, behavioral, and molecular landmarks have been described to significantly contribute in the understanding of mahi life development.

**Conclusion:**

Remarkably, despite the vast difference in adult size, many developmental landmarks of mahi map quite closely onto the development and growth of Zebrafish and other warm‐water, active Teleost fishes.

## Key Findings

- Mahi‐mahi is a tractable model for experimental studies high‐performance pelagic predatory fish species.
- Biological attributes of mahi are reported in a comprehensive developmental table.
- Physiological, behavioral and molecular landmarks are described through the life development.
- Mahi has a rapid growth rate, but the developmental marks are similar to other teleost fishes.

## 1 INTRODUCTION

*Coryphaena hippurus*, also known as the common dolphin fish or mahi‐mahi,^1^ is a highly migratory epipelagic fish distributed in the world's tropical and subtropical waters, where temperatures are typically between 25°C and 30°C.^2^, ^3^, ^4^  Mahi‐mahi (hereafter “mahi”) is economically important for recreational and commercial fisheries throughout this species' global range.^5^ Global capture was estimated to be 115 658 tons in 2014.^6^ The sustainable management, the circumglobal distribution, and the biology of mahi are attributes that make this species a viable choice for commercial aquaculture development.^5^, ^7^ Since the 1980s, technology for domestication of this species has been developed that allows mahi to be cultured successfully.^7^, ^8^, ^9^ However, full commercial‐scale productivity for aquaculture operations has yet to be demonstrated.

Aside from the interest in this species from a food‐fish perspective, mahi has been identified as a promising candidate for research investigations. Indeed, during the last few decades, mahi has become an emergent model for examining population genetics,^10^ developmental physiology,^11^, ^12^ metabolic responses,^13^ nutritional physiology,^7^ egg and larval performance over time,^14^ and climate change effects.^15^, ^16^ Mahi have also been studied extensively regarding the impact of environmental toxicants.^17^, ^18^, ^19^, ^20^, ^21^, ^22^, ^23^, ^24^, ^25^, ^26^, ^27^, ^28^, ^29^, ^30^ The emergence of this model fish has been accelerated by the need to develop more sophisticated scientific approaches for understanding the impact of environmental stressors, especially the impacts of the *Deepwater Horizon* Oil spill in 2010. Immediate mortality or fitness declines have been shown in fin fish populations as a result of pollution exposure (see numerous chapters in Burggren and Dubansky^31^). It has become crucial for the scientific community to use resident species from the Gulf of Mexico for assessing economic feasibility operations, and mahi is ideal for this task.

The biology of mahi provides some relevant benefits due to its fast growth rates, capacity of producing, high spawning frequency, and high reproductive energy allocation. Regarding numerous model fishes (eg, Zebrafish, medaka, trout), mahi shares several similar attributes including the ability for researchers to control the reproductive cycle to allow year‐round egg production^32^; the high number of produced eggs; and rapid embryonic development and transparency of embryos. These attributes make mahi a tractable model for future experimental studies.

Key to expanding studies on the developmental effects of the environment on mahi is a set of tools that includes a detailed table of development. Although a general developmental table documenting the main embryonic stages has been provided by Palko et al,^4^ it lacks numerous important details about morphological and physiological landmarks placed on a common timescale, and is mostly devoid of any behavioral or molecular data. Scattered observations currently exist in literature but have never been compiled into a comprehensive developmental table. Thus, in the present study, we have created a comprehensive developmental table for mahi based on the brood stock of the University of Miami Experimental Hatchery (UMEH). Rather than presenting a “conventional” (and somewhat tedious) table reporting development as primarily detailed observations of morphology, similar to that already available for numerous fishes,^33^, ^34^, ^35^, ^36^, ^37^, ^38^, ^39^, ^40^ in this study we have particularly stressed specific physiological, behavioral, and molecular traits from the zygote to adult stage, alongside the standard morphological changes.

## 2 RESULTS AND DISCUSSION

**Figure 1:**
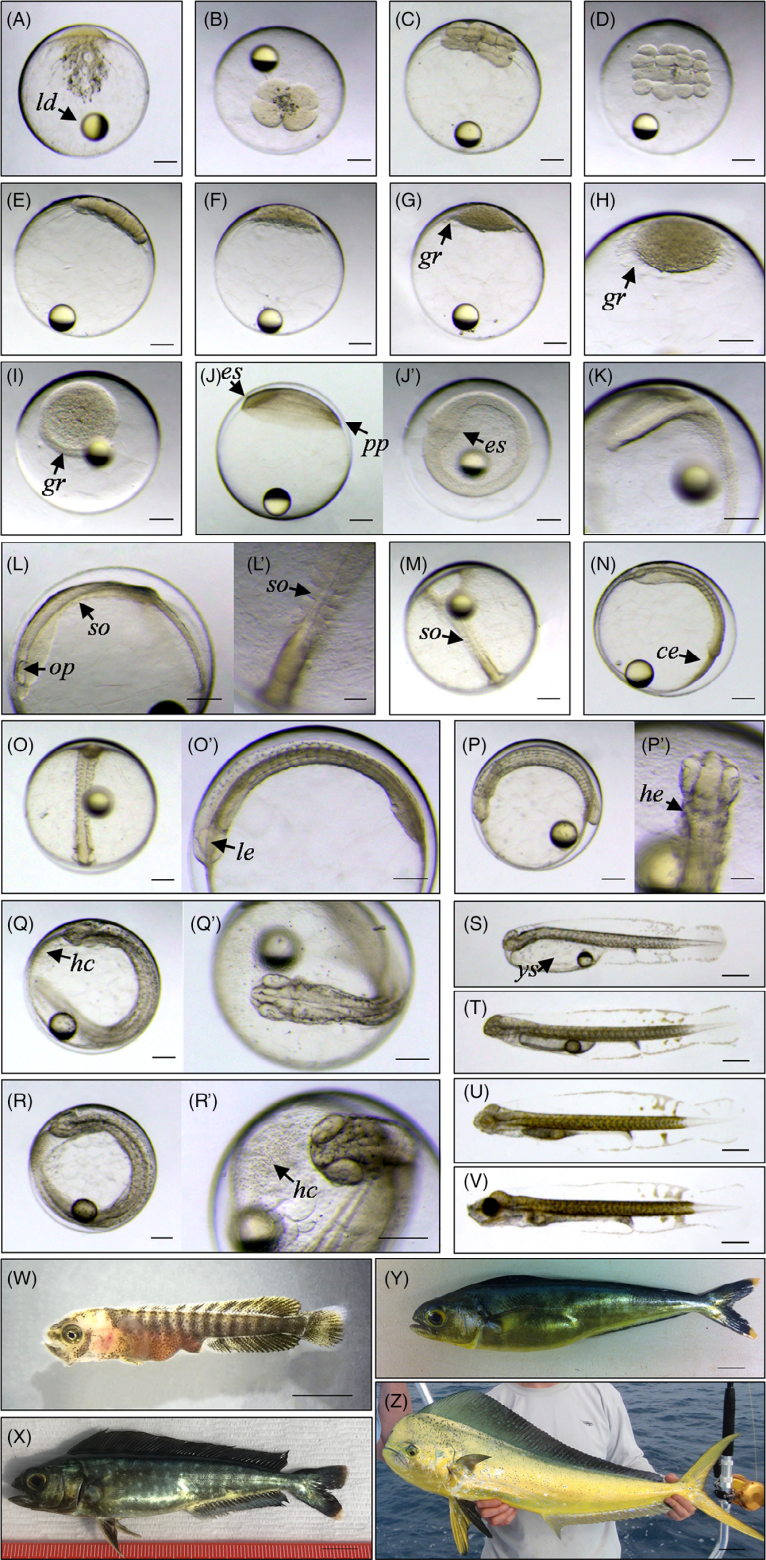
Embryo‐larval development of mahi at 26°C. A‐C, Cell cleavage. D‐I, Morula/blastula. J‐P, Gastrula/segmentation. Q–S, Pharyngula/hatching period. T–V, Post‐hatching period/yolk sac larvae. W–Y, Juvenile period. Z, Adult phase. A, Zygote stage, 1 cell (pre‐division, 0 hpf). B, 4 cells (second division, 50 mpf). C, 8 cells (third division, 70 mpf). D, 16 cells (fourth division, 80 mpf). E, 32 cells (fifth division, 120 mpf). F, 128 cells (seventh division, 3 hpf). G, 256 cells (eighth division, 4 hpf). H, 512 cells (ninth division, 5 hpf). I, Germ ring (6‐7 hpf). J, 20% epiboly (8 hpf). K, 50%‐60% epiboly (11 hpf). L, 80% epiboly, 3‐4 somites (14 hpf). M, 90% epiboly, 5‐6 somites (15 hpf). N, 100% epiboly, 8‐9 somites (16 hpf). O, 12 somites (18 hpf). P, 26+ somites (22‐23 hpf). Q, 35 hpf; R, Pre‐hatching period (38‐40 hpf). S, Hatched larvae (44 hpf). T, Yolk sac larvae (56 hpf). U, Protruding‐mouth stage (80 hpf). V, Mouth‐opening stage (104 hpf). W, Juvenile mahi (16 dph). X, 40 dph. Y, Transition to young adult phase (55 dph). Z, Adult male. ce, complete epiboly; dph, days post‐hatching; es, embryonic shield; gr, germ ring; hc, cells from hatching gland; he, heart; ld, lipid droplet; le, lens; op, optic primordium; pp, posterior pole; so, somites; ys, yolk sac. Scale bars A‐L,M‐P,Q‐R = 250 μm. Scale bars L′, P′, R′ = 100 μm. Scale bars S‐V = 500 μm. Scale bars W‐Y = 1 cm. Scale bar Z = 10 cm A–V: Photo credit: P. Perrichon; W–Z: Photo credit: J. D. Stieglitz

Following fertilization, mahi eggs are approximately 1.2 to 1.6 mm in diameter (Figure 1A). The eggs contain a single oil globule measuring 257 to 307 μm in diameter, depending on the captivity time.^14^ While no standardization exists in the embryogenesis staging of teleost fishes, major molecular and cellular processes that underlie early development are highly conserved across teleost fishes. Though many fish species share common features, not unexpectedly numerous differences also emerge among species, especially regarding staging timing and organ implementation or progression. Following fertilization, mahi grow rapidly^41^ compared to other model fishes. The most striking feature is the range of size that a mahi can reach in the first year compared to other model fishes (Table 1). Mahi are capable of growing from a hatch length (standard length [SL]) of 3.7 to 3.8 mm at 40 hours postfertilization (hpf) to a reproductively mature fish of ~20 to 30 cm at 80 to140 days postfertilization (dpf), while the maximum size of adult fish (length: 1‐2 m) is reached after 3 to 4 years. Longevity of mahi is particularly short compared to other pelagic species, with an average of 2 years and maximum of 4 to 5 years.^2^, ^4^

**Table 1:** Morphological, physiological, behavioral and molecular landmarks thought the life development of mahi

DEVELOPMENT			Phenotype							
Stage		Hours (unless otherwise indicated^4^)	Morphology					Physiology	Behavior	Molecular Biology
			General Observations	Cell Number (Division)	Length (mm)	Epiboly (%)	Somite (#)			
**Embryo**	Zygote	0	Spawning and fertilization typically occur at 2 am‐5 am 14 Eggs positively buoyant	1 (pre‐division)						
	Cleavage	35‐40 min	First cleavage	2 (1st division)						
		50‐70 min	Discoidal Meroblastic cleavage in blastodisc Greatest concentration of yolk at animal pole due to inhibition of cleavage at vegetable pole	4, 8 (2nd and 3rd divisions)						
	Morula/Blastula	80 min	Blastomeres evident Cell migration begins	16 (4th division)						
		120 min	Cells irregular in shape and arrangement	32 (5th division)						
		150 min		64 (6th division)						
		3	7th cleavage creates blastula with well formed blastodisc A primarily yolk‐filled blastocoel	128 (7th division)						
		4	Spreading of yolk syncytial layer	256 (8th division)						
		5	Yolk sac syncytial layer evident	512 (9th division)						
		6	Yolk syncytial layer Enveloping layer and deep cell layer increasingly distinctive	1024 (10th division and beyond)						
		6‐7	Germ ring formation			0				Zygotic gene transcription begins Heavy RNA transcription
	Gastrula	8	Onset of epiboly Spreading of yolk syncytial layer and blastoderm over and across yolk sac First appearance of embryonic shield			20				
		11	Spread of embryonic shield Invagination/involution to form mouth, anus, and digestive tube			50	0	Urea and ammonia excretion measurable^42^ Urea excretion rises sharply^42^		
	Gastrula‐Segmentation Transition	13 (12)	Appearance of neural groove Appearance of first somites		1.3‐1.5	70	1‐2			
		14	Otic placode evident Optic primordium appears without pigmentation		1.5	80	3‐4			
		15	Kupffer's vesicle formation at tail bud end Optic primordium evident		1.6‐1.7	90	5‐6			
		16	Blastopore closes to complete epiboly and signal full transition to segmentation phase Tail bud appears Early body pigmentation		1.7‐1.8	100	8‐9			
	Segmentation	17 (*17‐18*)	Yolk sac pigmentation appears, followed by start of spread to body surface		1.9‐2.0		10			Urea transporter gene starts to express
		18	Cardiac precursors and myocytes visible Myotomes begin to form in previously formed somite Embryo trunk curved ~50% of egg capsule diameter		1.9‐2.0		12			
		185 (*17*)	Somite number continues to increase		2.1‐2.5		13‐14			
		19 (*18*)	Cardiomyocytes visible Retina present Otic vesicle forms from otic placode Pronephric duct		2.2‐2.6		16‐17	No peristaltic heartbeat		
		22	Heart tube rudiment in anterior position, close to eye Otoliths evident Neural tube forming		2.6‐2.8		26+	Heartbeat onset as irregular peristaltic movements Skeletal muscle contraction initiated		
	Pharyngula	23 (*26*)	Maximum somite numbers achieved Heart tube elongating		2.8‐3.0		30‐34	Rudimentary sensory reflexes	Coordinated body movement begins Spontaneous weak embryo twitches within egg capsule	
		24	Embryo trunk curved 50%‐60% of diameter of egg capsule Melanophores numerous on body Single dorsal/caudal fins begin development, but tail still attached to yolk sac, which is large and conspicuous					Heart rate at 90‐95 beats/min^−1^	Intensification of body movements Reflex movements from touch	Expression of genes involved in general developmental biology (eg, cellular development, tissue development, organ development)^30^
		26‐27 (*26*)	Embryo trunk curved 60%‐70% of diameter of egg capsule Heart chambers not yet clearly delineated but discernable in videos of contracting heart atrium anteriorly located Elongated caudal fin starts to migrate off yolk sac		3.0‐3.2			Regular heart rate at 120‐130 beats/min^−1^		
		32	Heart still in embryonic configuration but greater delimitation of cardiac chambers Embryo position changing within yolk sac		3.4‐3.5			Vigorous cardiac contraction Heart rate^12^ increases to 160‐165 beats/min^−1^		
		34	Cardiomyocytes dividing to create thickening heart walls		3.7‐3.8			Oxygen consumption^24^ ~30‐35 PMol/ind^−1^/min^−1^		
		37‐38	Early pigmentation in eye, which remains largely transparent Caudal fin pigmentation starts (peripheric pigmentation)		3.7‐3.8			Urea excretion continues to increase^42^ Oxygen consumption increases sharply^24^	Frequent embryonic movement within egg 60%‐75% of eggs are negatively buoyant	Urea excretion rate and urea transporter gene expression reaches peak level around 36 hpf and steadily decreases thereafter^42^
		39	Prominent cells (granules) of hatching gland, present on pericardium over the anterior yolk sac Chorion begins to weaken No blood circulation						95%‐100% eggs are negatively buoyant	
**Hatching Embryo**		41	Earliest embryos hatch Pigmentation abundant in dorsal part Peripheral pigmentation in caudal, dorsal, and ventral Large pericardium and advanced in anterior position Pectoral fins rudiment visible		3.7‐3.8			2%‐5% of eggs hatched	Swimming reflex	
		42	Major veins apparent, no blood circulation		3.9‐4.0			10%‐20% of eggs hatched		Ammonia transporter gene (*rhag* and *rhbg*) expression significantly increase^42^
		43	Erythropoiesis initiation evident from appearance of transparent circulating blood cells Central circulation apparent as modest flow Major arteries and veins evident		3.9‐4.0			40%‐45% of eggs hatched Peristaltic blood flow through cardiac chambers well established Complete constriction between atrium and ventricle during cardiac cycle		
		44	Circulating in major arteries and veins Very low blood cells circulation		4.0‐4.1			80%‐86% of eggs hatched		
		45	Last embryos hatch		40‐41			>97% of eggs hatched		
**Free‐swimming Larva**	Yolk Sac	42‐46	Peripheral fin pigmentation Pectoral bud starts to develop (shallow dome) Large pericardium Atrium larger than ventricle Body curvature still apparent							Ammonia excretion rate increases significantly
		48‐49	Trunk and peripheral fin pigmentation increasing Pectoral bud increases in size (height dome and bud curve) Ventral fin well developed Intensification of body pigmentation and eye Brain area and tail end still relatively transparent Reduction of pericardium volume Head and body are aligned Retinal pigmentation appearing		4.2‐4.3			Heart rate at 175‐180 beats/min^−1^	Larvae are vertically oriented in water	Expression of genes involved in RNA binding, ATP binding, neurogenesis, and development of cardiovascular, visual, and muscular systems^30^
		50‐51	Cardiac chambers well differentiated Heart begins S‐folding Oil droplet persistent Pectoral fins continue to develop		4.3‐4.5			Cardiac chambers (including bulbus arteriosus) beat in a coordinated sequence Arterial branchial and systemic circulation established	Sporadic, “twitch‐like” swimming	
		56	Atrium, still larger than ventricle, positioned dorsal to ventral Bulbus cordis and sinus venosus become differentiated Eye pigmentation clearly observed but still light Pectoral fin well developed but still “attached” to the body Stereocilia of anterior neuromast well developed Faster blood flow through body but blood cells still discernable		4.5‐4.6			Rudimentary valving action evident between contracting and relaxing heart chambers Heart rate^11^, ^26^ at 190‐195 beats/min^−1^ Cardiac output^11^, ^26^ at 27‐29 nL/min^−1^ Oxygen consumption^24^ at ~250‐275 PMol/ind^−1^/min^−1^		*Rhag* and *Rhbg* expressed in gills^42^ *Rhag* expressed in yolk sac^42^ *NHE3* expressed in heart and intestines^42^
		80	Protruding mouth Pectoral fins continue elongation Movement of pectoral fins start Cardiac ventricle growing in size relative to atrium		4.8‐4.9				Larvae are not vertically oriented in water anymore Swimming behavior increases, but fin movement limited	
		104	Mouth open Eye pigmentation intensifies Yolk sac absorption nearly complete Branchial structures (gill arches and operculum) largely formed		4.9‐5.0				Active pectoral fins movement First exogenous feeding Vision‐dependent behavior [JTM, unpublished data]	Rhch2 expressed in skin^42^ Expression of genes involved in metabolism functions (eg, lipid metabolism, carbohydrate metabolism), RNA binding, ATP binding, cellular catabolic process, metabolism, neurogenesis, development of cardiovascular, visual and muscular systems^30^
	Post‐yolk Sac Absorption	120‐128	Retina, lens, ganglion, epithelium, plexiform, and photoreceptor layer anatomically distinct		4.9‐5.0			Irregular buccal and opercular pumping creating gill ventilation at ~70‐90 movements/min^−1^ [PP, unpublished data]		
		152			4.9‐5.0			Regular buccal and opercular ventilation at 100‐110 movements/min^−1^ [PP, unpublished data]		
		176	Dorsal, caudal, and anal fins still a single elongated fin Yolk sac resorbed		5.1‐5.2					
**Juvenile**		10 days	Flexion stage^26^						Larva responsive to changes in surrounding visual field [JTM, unpublished data]	
		11‐13 days	Postflexion^26^						Visual field sensitivity increases [JTM, unpublished data]	
		15 days	Adult fin configuration Body pigmentation and coloration of alternating dark and light bars Blunt snout characteristic of adults appearing Eyes and mouth fully developed Musculature evident		15			Olfactory response to environmental components evident	Negative chemotaxis	
		18 days	Transition from pale brown/yellow coloration to darker brown/black coloration (fish gain ability to manipulate color phase at this point, particularly evident during feeding events)							
		20 days	Caudal fin shape transition begins from rounded (pre‐20 days) to emarginate (21‐ ~55 days) and then eventually to forked at ~55+ days							
		26 days	Body mass 200‐300 mg		33‐35					
		32 days	Body mass 600‐700 mg Lateral banding on body less distinctive than earlier juvenile states Caudal fin begins to develop deep fork characteristic of adults		42‐46			Standard O_2_ consumption^28^ at 1.0 mg O_2_/g^−1^/h^−1^ U_crit_ at 4.8‐5.0 body length/sec^−1^ 21, 28	Cannibalism continues throughout juvenile and adult life	
		45 days	Body coloration transitions from primarily darker phase (dark brown/black) to a more silvery/reflective phase Upon transition to the juvenile stage with silvery appearance, whereby stressed individuals will express the “classic” yellow‐green coloration typically associated with mahi‐mahi, the fish will begin schooling behavior						Classic mahi‐mahi yellow/green coloration when stressed	
		55 days	Tail fully forked							
**Adult**		80‐90 days	Sexual maturity reached by earliest maturing individuals under optimal rearing conditions		20‐30 cm					
		120‐140 days	Body mass 255‐301 g Sexual maturity reached by majority of population^32^, ^42^		~30 cm			Heart rate^23^ at 140‐164 beats/min^−1^ Cardiac output^23^ at 50‐60 mL/min^−1^/kg^−1^ Standard O_2_ consumption at 490‐500 mg O_2_/kg^−1^/h^−1^ U_crit_ at 4.0‐4.2 body length/sec^−1^ 7, 21		
		4‐5 years	Maximum age and size acquired Up to 40 kg (IGFA record)		1‐2 m					

hpf, hours postfertilization.

As a whole, total embryogenesis from fertilization to the first exogenous feeding stage lasts for 104 hours at 26°C (Table 1 and Figure 1). Despite the size difference, this developmental duration for embryogenesis is somewhat comparable to that of Zebrafish (120 hpf at optimal temperature of 28°C). Other model fishes exhibit longer embryonic development: 9 to 11 days for *Oryzias latipes* (medaka; 6°C), 10 to 12 days for *Fundulus heteroclitus* (killifish; 20°C), 34 to 37 days for *Oncorhynchus mykiss* (rainbow trout; 10°C), and 15 days for *Perca fluviatilis* (Eurasian pikeperch; 13°C) (Table 2).^33^, ^34^, ^36^, ^40^ In mahi, the first period of cleavage occurs at 35 minutes postfertilization (mpf) and results in two blastomeres of equivalent size, as observed in most teleost fishes. Cell division continues (Figure 1B,C), and cell migration begins at 80 mpf (16 cells; Figure 1D). A blastula with a well formed blastodisc appears at 3 hpf (128 cells, seventh division; Figure 1F–H). At 6 to 7 hpf, the germ ring is well defined (Figure 1I) and the activation of zygotic gene transcription accompanied by extensive RNA transcription occurs. The first epiboly movements then begin. From 8 hpf, gastrulation takes place with the appearance of the embryonic shield (Figure 1J). Physiologically, urea and ammonia excretion have been measured during early gastrulation^42^ (Table 1).

**Table 2:** Comparative life characteristics between mahi and three model fish species

Characteristics	Mahi‐mahi^3^, ^4^, ^32^ (*Coryphaena hippurus*)	Zebrafish^37^, ^43^ (*Danio rerio*)	Killifish^34^ *(Fundulus heteroclitus, F grandis*)	Rainbow Trout^40^ *(Oncorhynchus mykiss)*
***Habitat***	Wildly distributed, offshore (tropical and warm temperate waters)	Central Asia, India, Ganges River	Inshore bays, salt marsh flats, estuaries and tidal creeks with emergent vegetation	Wildly distributed (cold waters)
***Economic interest***	Sport fish	Ornament fish	Ornament fish	Sport fish
***Swimming capacity***	Fast swimmer (migratory fish)	Low swimmer	Low swimmer	Variable (migratory fish)
***Lifespan***	4‐5 y	4‐5 y (mean 3.5 years)	4‐5 y	6‐8 y (11 y record)
***Adult morphometry***	1‐2 m, up to 40 kg (IGFA record)	4‐5 cm	5‐10 cm (15 cm max)	50‐80 cm
***Environment***	Saltwater	Freshwater	Fresh, brackish, and saltwater	Fresh, brackish, and saltwater
***Temperature tolerance***	19°C‐31°C	25°C‐31°C	6°C‐35°C	0°C‐27°C
***Embryonic rearing condition***	Air incubation	Static water	Air incubation until hatching	Running water
***Breeding season***	Year‐round	April to August (year‐round in laboratory)	March to September (semi‐lunar rhythm, year‐round in laboratory)	March to July
***Breeding facilities required***	80 000 L tank	1‐150 L tank	20‐150 L tank	30 000 L tank
***Cost level (relative units)***	++++	+	++	+++
***Spawning size***	100 000‐200 000 eggs per female (3‐5 kg)	100‐500 eggs per female	200‐400 eggs per female	1500‐2000 eggs per kg
***Egg buoyancy***	Positive until 33‐40 hpf	Negative	Negative	Negative
***Chorion strength***	Strong	Weak	Thick, strong	Thick, strong
***Egg size at spawning***	1.2‐1.5 mm	1.0‐1.2 mm	2.0‐2.3 mm	3.5‐5 mm
***Egg incubation period***	Very short	Short	Medium (aerial incubation)	Long
***Hatching time (postfertilization)***	41‐45 h (26°C)	48‐72 h (28°C)	10‐12 d (20°C)	34‐37 d (10°C)
***Epiboly process***	7‐16 h (“Gastrulation + segmentation transition,” germ ring to tail bud apparent, 8‐9 somites)	4.25‐10 h (“Gastrulation” dome stage to bud, no somite)	30‐46 h (“gastrulation,” somite formation onset)	5‐9 d (“Gastrulation + segmentation,” germ ring to 21‐29 somites)
***Heartbeat onset***	22 h	24 h	85‐92 h	14‐15 d
***Protruding mouth***	75‐80 h	72 h	9‐10 d	18 d
***Feeding initiation***	104 h	120 h	10‐12 d	34‐37 d
***Complete yolk sac absorption***	7 d	7 d	16 d	85 d
***Sexual maturation***	80‐90 d	90 d	9 m	2 y

hpf, hours postfertilization.

One notable feature during mahi embryonic development is that the segmentation process/somitogenesis overlaps with the epiboly process (Figure 1J–N). The cellular front reaches 50% epiboly at 11 hpf (Figure 1K) and 70% of the yolk surface by 12 to 13 hpf, corresponding to the formation of the first somites. At the beginning of the somitogenesis, mahi embryos are 1.3 to 1.5 mm in length. Complete epiboly is reached at 16 hpf (8‐9 somites; SL = 1.7‐1.8 mm; Figure 1N). The somites progressively increase in number during the segmentation process (17‐22 hpf; Figure 1O,P). The epiboly process also overlaps with somitogenesis in rainbow trout (3‐9 dpf, until 29 formed somites) (Table 2).^40^ While the epiboly and segmentation steps progressively succeed one another in Zebrafish, the timing of morphogenesis and cardiogenesis is very similar in both Zebrafish and mahi (Tables 1 and 2).^37^

At the beginning of the segmentation period in mahi, early body pigmentation and individual cardiomyocytes are visible. Urea transporter genes also begin their expression.^42^ At 22 hpf (SL = 2.6‐2.8 mm; 26 somites; Figure 1P), the onset of the heartbeat begins with irregular peristaltic movements, which closely resemble those of the Zebrafish embryo, where heartbeat occurs at ~24 hpf. At this stage, expression of genes associated with cellular, tissue, and organ development is also accelerating.^30^ The first muscle contractions of mahi embryos are also observed at this time in development, and embryonic movements sharply increase with a touch reflex apparent from 26 hpf. Regular heart rate (120‐130 beats/minute^−1^) is established by 26 hpf in mahi, whereas heart chambers are discernable but not yet delineated. Heart rate frequency then increases with further development.^11^, ^12^ Urea transporter gene expression peaks around 36 hpf.^42^ Erythropoiesis is initiated from transparent circulating blood cells, and circulation in the central vasculature appears as a modest flow during hatching (43 hpf) (SL = 3.9‐4.0 mm). At this point in development, ammonia transporter (Rhag and Rhbg) gene expression increases as urea transporter expression decreases.^42^ Complete morphological constriction between the atrium and ventricle is seen during the hatching period, and the heart (including bulbus arteriosus) initiates its S‐folding configuration at 50 hpf (SL = 4.3‐4.5 mm). Concurrently, arterial branchial and systemic circulation are established. Oxygen consumption (cutaneous respiration or simple diffusion) is measurable in early stages from 34 hpf (Table 1).

Mahi eggs are positively buoyant in the laboratory and are assumed to float near the surface of the water column in the field. Prior to hatching, egg specific gravity changes, cells (granules) of the hatching gland increase in number over the anterior yolk sac (Figure 1Q–R’), and the eggs become negatively buoyant until they hatch.^27^ This dynamic change in buoyancy occurs 2 to 4 hours before hatching in mahi, and has also been observed in tuna species.^44^ Buoyancy changes and the process of sinking in the water column prior to hatching likely reduce mortality of newly hatched larvae exposed to wave action and wind, while further minimizing the exposure to UV radiation and predation at the surface.^27^

The hatching period lasts for several hours (40‐45 hpf), and individuals hatch into relatively undeveloped yolk sac larvae (SL = 3.7‐4.1 mm) lacking a functional mouth, eye pigmentation, and differentiated fins (Figure 1S). For comparison, Zebrafish hatch between 48 and 72 hpf. Accelerated expression occurs in genes involved in RNA binding, ATP binding, neurogenesis, and development of cardiovascular, visual, and muscular systems.^30^ Following hatching, the pectoral buds, trunk, and peripheral fins continue to develop (Figure 1S–V). Movement of pectoral fins starts at 80 hpf (Figure 1U) along with increased swimming behavior. A protruding mouth appears at 80 hpf, and the first exogenous feeding starts from 104 hpf (SL = 4.9‐5.0 mm; Figure 1V), where vision‐dependent behavior is activated to strike planktonic prey in the water. The first oral feeding by larvae occurs relatively early compared to model fishes such as the Zebrafish (120 hpf), killifish (10‐12 dpf), and rainbow trout (34‐37 dpf). At 80 hpf in mahi development (Figure 1V), branchial structures are largely formed and opercular pumping, creating gill ventilation (70‐90 movements/minute^−1^), begins around 120 hpf and steadily increases in frequency and depth with additional development. Complete absorption of yolk sac occurs by 176 hpf, similar to Zebrafish.

In parallel to morphological, physiological, and developmental changes observed early in development, the expression of genes and regulation pathways reveals a transitional state related to the described physiological and behavioral changes during development (Table 1).^30^ High‐throughput sequencing demonstrates that a significant contribution of genes is involved in cellular and tissue development from the pharyngula period (~24 hpf) to yolk sac stage (~48 hpf). Additionally, metabolism‐related processes are more enriched during development of free‐swimming larvae and associated with cardiovascular, muscular, and neuronal development.^30^

Increased retinal pigmentation is observed at first feeding, with distinct retinal lamination (lens, neuronal layers, photoreceptors) observed by 5 dpf. Vision‐dependent behavior is increased by 10 dpf along with an increase in the sensitivity of mechanical stimulation. This vision‐dependent behavior highlights a gradual transition stage from the larval to juvenile state (SL = 15 mm at 15 dpf).

At this point (10 dpf), mahi enter the flexion stage, where musculature is evident throughout the body and the eyes and mouth are prominent and fully developed. Fin development progresses (Figure 1W) and displays the adult configuration from 30 to 40 dpf. Fishes reach adult configuration (body coloration and fin formation) from 40 to 55 days (Figure 1X–Y) and are sexually mature from 80 to 90 days under optimal rearing conditions (SL = 20‐30 cm).

In summary, mahi are large pelagic fish (Figure 1Z) with high energetic requirements necessary to maintain their “high‐performance” lifestyle.^7^, ^23^, ^28^, ^32^, ^42^, ^45^ Their physiological and metabolic capacities are therefore elevated for the increased supply of energy, oxygen, and substrate needed for swimming performance and routine activities^23^, ^28^ compared to those of more established fish models (eg, killifish, medaka, Zebrafish). From a developmental perspective, mahi share numerous physiological and behavioral landmarks with others pelagic fish (tunas or billfishes) as a result of a similar lifestyle.^45^ Perhaps most surprising is how closely the development of mahi compares with that of Zebrafish. We particularly hope that the attractive advantages of the mahi embryos will entice the scientific community to work on this biological system in the near future.

## 3 EXPERIMENTAL PROCEDURE

### 3.1 Fish populations examined

The developmental table in this study is based on the resident populations at UMEH. Mahi brood stock were captured in the offshore waters of the Strait of Florida off the coast of Miami, Florida, in the general coordinates of 25° 34.000′N / 80° 00.000′W using hook‐and line‐angling. Brood stock age and growth metrics, as well as methods of capture, transport, acclimation, and spawning, have been detailed in Stieglitz et al.^32^ The adult fish were subsequently transferred to UMEH, where they were acclimated in 80‐m^3^ fiberglass maturation tanks equipped with partially recirculated and temperature‐controlled water at 26°C to 27°C.^32^ All fish were regularly fed rations of whole and chopped Spanish sardines (*Sardinella aurita*) and squid (*Loligo opalescens*) to satiation every day. The brood stock were also fed with a supplement dry pelletized diet (MadMac‐MS, Aquafauna Bio‐Marine, Inc., Hawthorne, CA) once a week at 10% of the food weight per day. The nutritional composition of the natural diet and dietary supplements is further described in Stieglitz et al.^7^

Spawning occurred volitionally (noninduced) at a sex ratio of 1 male:8 females using standard UMEH methods.^14^, ^32^ Spawning events occur naturally throughout the year at UMEH between 2 am and 5 am before sunrise. Brood stock spawned naturally every day, with multiple females spawning asynchronously on opposing days.^14^, ^32^ Spawning patterns are relatively time‐specific in order to maintain consistent hatching periods (during the night), which is thought to maximize early larval survival. This adaptive spawning pattern has also been observed in other tuna species.^44^, ^46^

### 3.2 Data collection

Embryos were immediately collected after spawning events and were equally distributed among 1‐L glass beakers, where they were kept under optimal rearing conditions (26°C; 34‐35 ppm; photoperiod, 16 hours:8 hours light:dark). Early embryogenesis in fish was followed from the zygote stage (0 hpf; 1 cell) to post‐yolk sac absorption (176 hpf) by examining embryos under a Nikon SMZ‐800 stereomicroscope coupled to a Fire‐i400 or Fire‐i530c digital camera (Unibrain, San Ramon, CA). Observations from juvenile to adult stages were made directly from the production tanks. Major developmental landmarks and morphology of specimens were observed; images were digitized using Photo Booth software (dslrBooth Lumasoft, East Brunswick, NJ) and calibrated using a stage micrometer. ImageJ software^47^ was used then to measure specific physiological parameters such as larval cardiac output.

All developmental and phenotypic observations are normalized as minutes, hours, or days postfertilization (mpf, hpf, and dpf, respectively) and are reported in Table 1. Although the intent of this study is not to describe a detailed pictorial representation of mahi development, Figure 1 illustrates the major developmental stages. Physiological, behavioral, and molecular characteristics were aggregated from data acquired during the last three years of research from the RECOVER consortium, supported by the Gulf of Mexico Research Initiative, involving four American universities (University of Miami, University of North Texas, University of California Riverside, and University of Texas Austin Marine Research Institute). Different rearing conditions, fish size, and/or nutritional status may influence the timing, developmental progress, and organogenesis of specimens.

## CONFLICT OF INTERESTS

The authors declare no competing financial interests.
